# Exploring Global Exposure Factors Resources for Use in Consumer Exposure Assessments

**DOI:** 10.3390/ijerph13070744

**Published:** 2016-07-22

**Authors:** Rosemary T. Zaleski, Peter P. Egeghy, Pertti J. Hakkinen

**Affiliations:** 1 ExxonMobil Biomedical Sciences, Inc., 1545 Route 22 East, Annandale, NJ 08801, USA; 2 Office of Research and Development, National Exposure Research Laboratory, U.S. Environmental Protection Agency, Research Triangle Park, Durham, NC 27711, USA; egeghy.peter@epa.gov; 3 National Library of Medicine, National Institutes of Health, 6707 Democracy Boulevard, Suite 510, Bethesda, MD 20894, USA; pertti.hakkinen@nih.gov

**Keywords:** consumer products, exposure factors, databases, exposure assessments, online, time-activity patterns, consumer behavior, household products

## Abstract

This publication serves as a global comprehensive resource for readers seeking exposure factor data and information relevant to consumer exposure assessment. It describes the types of information that may be found in various official surveys and online and published resources. The relevant exposure factors cover a broad range, including general exposure factor data found in published compendia and databases and resources about specific exposure factors, such as human activity patterns and housing information. Also included are resources on exposure factors related to specific types of consumer products and the associated patterns of use, such as for a type of personal care product or a type of children’s toy. Further, a section on using exposure factors for designing representative exposure scenarios is included, along with a look into the future for databases and other exposure science developments relevant for consumer exposure assessment.

## 1. Introduction

Assessing consumer product-related exposures has been of global interest and effort for several decades, with many key moments of progress, such as the publication of the U.S. EPA’s first edition of the “Exposure Factors Handbook” in 1989 and the multi-organizational global effort to publish the seminal sourcebook on residential exposure assessment in 2001 [1,2]. Today’s consumer products contain and potentially release an array of chemicals, including substances such as engineered nanomaterials. Consumers and bystanders, such as children, may be exposed to these substances through direct or indirect dermal, oral and/or inhalation contact [3,4,5,6,7]. There is now greater public interest and awareness in understanding exposures associated with consumer product use, partly as a result of greater public access to related information on the Internet, as well as from the continued expansion of human biomonitoring surveys of population exposure to chemicals from all sources, including consumer products [8,9,10]. Furthermore, regulatory and legislative efforts, such as the European Union’s REACH (Registration, Evaluation, Authorisation and Restriction of Chemicals), legislation have created a greater awareness of, and efforts toward, assessing exposures in the residential environment [11,12,13].

Three types of products are particularly relevant for exposures in residential environments, namely: consumer products, household articles and building materials. Of these, the greatest amount of publicly-available compositional information is for consumer products, such as cleaning products and personal care products, with ingredients often available on the labels of the individual products, on the websites of formulators and retailers, and increasingly in publicly-available databases [14,15,16]. Information on the weight fraction of ingredients in a consumer product remains more limited. Since human exposures are strongly influenced by the ways in which the materials and products are used, information on consumer behavior is equally important as chemical composition [17]. A number of the most comprehensive studies on consumer product use are somewhat dated surveys (1987) with a narrow focus (e.g., household cleaning products, solvents and paints) [18,19,20]. More recent studies (2005–2013), although often smaller in scale, have investigated usage patterns of household and personal care products, as well as the chemical ingredients of these products [21,22,23,24,25,26,27,28,29].

Worldwide interest in conducting scientifically-relevant exposure and risk assessments for chemicals found in consumer products underscores the need for increased access to informational resources for understanding the key determinants of exposure based on product use characteristics and consumer behavior. The purpose of this document is to summarize current exposure factor information relevant to consumer exposure assessment and to provide a resource for the exposure assessment community seeking exposure factor data useful for consumer exposure assessment. Exposure factors relevant for consumer (or residential) exposure assessment cover a broad range, from general physiological parameters, such as inhalation rate or skin surface area, to product-specific use behaviors, to parameters that help characterize the indoor environment.

Our approach to provide some organization around these data sources was to summarize the resources into general exposure factor sourcebooks (each covering multiple parameters), sources that focus in greater detail on one particular type of exposure factor and product-specific exposure factors organized by product category. When a source spans multiple types of information, it is likely to be cited multiple times. We have not attempted to summarize the detailed data included in these sources here, but rather provide the types of information that may be found in each source. Our intent is not to perform a comparison across sources or a quality assurance check on each one, but is simply to increase the awareness of available information. Given the range in time period and population geography spanned in the studies cited, considerations as to the likelihood of regional or temporal variation in a particular exposure factor should be taken into account when applying the study data (hyperlinks to the identified resources when available are included within the text; a compiled list of these links is also provided in Supplemental Materials Table S1).

## 2. Identified Resources

### 2.1. General Exposure Factor Data Resources: Published Compendia and Databases

There are several key sources of general exposure factor information, well recognized and utilized within the field of exposure assessment. In this section, a brief overview of the information found in each is provided. These information sources may include general exposure factors and product-specific information. Where product-specific information is included, these same sources are discussed in more detail under the product specific section. Table 1 provides a summary of the type of exposure information associated with many of these key references.

#### 2.1.1. North America

- The U.S. EPA “Exposure Factors Handbook” (EFH) (2011) contains general information for the U.S. population [30]. Exposure factors include anthropometric data (e.g., body weights, skin-surface areas and life expectancy), behavioral data (e.g., activity/time use patterns, consumer product use), physiology (e.g., inhalation rates, dermal adherence factors), ingestion (drinking water consumption, soil ingestion, consumption of fruits and vegetables, fish, meats, dairy products, homegrown foods, human milk intake) and environmental (housing characteristics). Recommended values are for the general population and also for various segments of the population who may have characteristics different from the general population. Full distributional data are provided in the EFH when available, to support both probabilistic and stochastic exposure and risk assessment. It also includes product-specific information. The current version incorporates information from the previous “Child-Specific Exposure Factors Handbook” [31] and is meant to supersede the stand-alone child-specific version [30]. A related U.S. EPA effort led to the “Child-Specific Exposure Scenarios Examples” as a companion document to the EFH [32]. The example scenarios were compiled from questions and inquiries received from users of the EFH on how to select data from the EFH to assess childhood exposures.
- The “Residential Exposure Assessment Sourcebook” (2001) [2] was the result of a multiyear effort known as the Residential Exposure Assessment Project (REAP) initiated by the Society for Risk Analysis (SRA) and what is now the International Society of Exposure Science (ISES). Expert working groups were organized for each of the thirteen chapters to develop the content, e.g., chapters on consumer products, human exposure factors, residential exposure factors, assessment of dermal, oral and inhalation exposures in the residential environment, uncertainty analysis, communication of results, etc.
- “Toxicology and Risk Assessment” (2015) contains multiple chapters summarizing exposure factor information, primarily based on the U.S. population, with the U.S. EPA EFH serving as a key information source [33]. Chapters address exposure factors, such as age-specific physiology and time-activity patterns. The report also includes some information on consumer product usage, as well as food consumption.
- “Data Sources Available for Modeling Environmental Exposures in Older Adults” (2011) is an EPA report that discusses the information currently available on the unique factors that shape seniors’ exposure, in the context of providing inputs to EPA exposure models. It includes information on day-to-day habits, activity levels and the environments in which older adults typically live and work. Topics include anthropogenic and physiological values, energy expenditures and indices of physical activity, time use patterns and impairment and disability [34].
- “A review of physiological and behavioral changes during pregnancy and lactation: Potential exposure factors and data gaps” (2014) provides information on physiological and behavioral exposure factors that may change during pregnancy [35]. Exposure factors investigated include water ingestion, dietary intake, inhalation rate, location based activities, activities associated with exposure to water and the use of selected personal care products.
- The “Canadian Exposure Factors Handbook” (2013) was recently developed to replace the “Compendium of Canadian Human Exposure Factors for Risk Assessment” as a resource for risk assessments that reflect the Canadian population [36,37]. The data and statistics presented in this handbook are based on more current surveys that better reflect current Canadian population characteristics, behaviors and activities. The analyses and statistics are based on datasets from a variety of Statistics Canada surveys, including the 2000–2010 Canadian Community Health Surveys, the 2005 and 2010 General Social Surveys and the 2007 Canadian Health Measures Survey. Statistics are provided by age group and gender for life expectancy, body dimensions, inhalation rates, time-activity patterns and soil ingestion rates. Most notably, time-activity data have been significantly updated to reflect the increasing time spent by teens, adults and seniors in indoor sedentary activities.

#### 2.1.2. Europe

- The E.U. ExpoFacts database is an online database of exposure factors information for multiple E.U. populations, hosted by the European Commission’s Joint Research Centre (JRC) [38,39]. Parameters include physiology, dietary ingestion, non-dietary ingestion, time-activity, housing characteristics and population indices. ExpoFacts contains data from 31 European countries: Austria, Belgium, Bulgaria, Croatia, Cyprus, the Czech Republic, Denmark, Estonia, Finland, France, Germany, Greece, Hungary, Iceland, Ireland, Italy, Latvia, Lithuania, Luxembourg, Malta, The Netherlands, Norway, Poland, Portugal, Romania, the Slovak Republic, Slovenia, Spain, Sweden, Switzerland and the United Kingdom. The ExpoFacts database was a follow-up to the “Exposure Factors Sourcebook for European Populations (with a focus on U.K. data)” [40]. This document was an initial effort to collect exposure factor data (physiological parameters, time-activity patterns and receptor contact rates) specific to European populations. A section on good exposure assessment practices is also included in the document.
- The European Chemicals Agency (ECHA) “Guidance on Information Requirements and Chemical Safety Assessment” and Chapters R. 15 “Consumer Exposure Estimation and R. 17 Estimation of Exposure from Articles” (2012) aim to describe an efficient, step-wise and iterative procedure for the estimation of consumer exposure to chemicals on their own, in mixtures or in articles [41,42]. The guidance focus is on the European population. The document includes defaults for multiple exposure factors and also lists of exposure models.
- The “Existing Default Values and Recommendations for Exposure Assessment” (2012) by the Nordic Council of Ministers gives an overview of non-chemical-specific exposure factors to be used during the process of assessing exposure to both adults and children, as well as of risk assessment in relation to REACH [43]. Its purpose is to contribute towards a further harmonization of such exposure factors to be used in exposure assessments. The following non-chemical-specific exposure factors are addressed: body weight, body surface areas, inhalation rates, ingestion of drinking water, intake of food, ingestion of soil and dust, non-dietary ingestion factors, lifetime expectancy, activity factors and consumer products.
- The Dutch National Institute for Public Health and the Environment (RIVM) ConsExpo “General Fact Sheet” (2006, 2014) includes data aimed at the Dutch population, but with broader information considered [44,45]. The focus is on exposure parameters that can be applied in various exposure scenarios within the ConsExpo model to produce a quantitative estimate of exposure to substances in consumer products. The general fact sheet includes information on body surface area and housing characteristics (ventilation and room size). The 2014 update also includes data on activity patterns. The RIVM ConsExpo website also provides a set of product-specific fact sheets (as discussed later in this document), which have been periodically updated with new information. [46]
- The German Exposure Factors Database (RefXP) [47] contains exposure factor data for food and drinking water consumption, time-location and activity patterns, soil and dust ingestion, residential characteristics and anthropometrics for the German population. A major update and expansion of the database occurred in 2006. Reference values and distributions are provided in the database. Data descriptions are available in English, and the database can be downloaded free of charge.

#### 2.1.3. Asia

- The “Korean Exposure Factors Handbook” (2006–2014) provides information specific to the Korean population. It includes information for physiological factors, soil ingestion, food intake, time-activity patterns, life expectancy and housing characteristics [48,49]. The resource is available in Korean, with summaries available in English [50,51,52,53,54,55].
- The “Japanese Exposure Factors Handbook” (2006) Contains data for the Japanese population [56]. The full resource is available in Japanese; a summary table in English is also available. The summary table includes representative values of daily intake for food categories, including mother’s milk or formula for infants. Time-activity information, physiological factors and soil ingestion are also included, as described in Phillips and Moya (2014) [49].
- “Highlights of the Chinese Exposure Factors Handbook (Adults)” (2015) was developed by the Ministry of Environmental Protection of the People’s Republic of China. The recently-published “highlights” book provides a brief, but comprehensive understanding of the environmental exposure-related factors for the Chinese population based on data from a national survey conducted by the Chinese Research Academy of Environmental Sciences [57]. Each chapter provides definitions, influencing factors and recommended values (at the mean, median, P5, P25, P75 and P95), stratified by urban/rural areas, regions, gender and age groups. The handbook covers inhalation rates, water and food ingestion rates, time-activity factors, anthropometric reference data and residential factors.
- The “Australian Exposure Factor Guide” [58] serves as a companion to the 2012 update of the Australian Environmental Health Standing Committee guidance document “Environmental Health Risk Assessment: Guidelines for assessing human health risks from environmental hazards” [59]. As with similar documents from other countries, the handbook is intended to provide risk assessors with sets of tabulated data on human factors that may be used as inputs to the exposure assessment component of an environmental health risk assessment. Australian exposure factor information is juxtaposed with overseas data, revealing that some overseas data may not reflect sectors of the current Australian population. The guide includes data for exposure factors related to physiology, both dietary and non-dietary ingestion, housing characteristics and time-activity patterns.

#### 2.1.4. International and Nonspecific

- Exposure factors resources: contrasting EPA’s Exposure Factors Handbook with international sources provides a general overview of the contents of the EPA’s and comparisons with other selected international exposure factors resources (including the Korean and Japanese Handbooks, the Australian Guide and the European ExpoFacts Database described above). Recommended values are compared across countries [49]. It identifies those exposure factors that are similar across most international populations because of their relationship to the physiological requirements of the human body (e.g., total water intake) and those that differ because they are influenced by dissimilar geographical, cultural or social factors (e.g., time-activity patterns, food preferences and intake rates). The authors identify areas where international standardization of methods for collecting and reporting exposure factor data can be beneficial to increase the overall data pool and leverage limited resources.
- U.S. EPA and the International Life Sciences Institute (ILSI) Databases for Physiological Parameters for Physiologically-Based Pharmacokinetic (PBPK) Modeling (2009) represents combined information from the initiatives of ILSI and the U.S. EPA. An ILSI work group developed a Database of Physiological Parameters for Early Life Stages, which has since been incorporated into the U.S. EPA’s downloadable Physiological Information Database (PID) [60]. EPA’s PID “contains physiological parameter values for humans from early childhood through senescence as well as similar data for laboratory animal species (primarily rodents).” These databases represent a readily-available compendium of the types of detailed internal physiological data that are needed to move from external to internal target organ exposure estimations. While a detailed review of internal physiological exposure factor data relevant to PBPK modeling was not conducted for this document, the utility and growing need for this type of information should be recognized.
- The International Commission on Radiological Protection (ICRP) “Report of the Task Group on Reference Man” (1975) was intended primarily for health physicists in making calculations of doses from internally-distributed radionuclides and to help in distinguishing experimental causes of differences in dose estimates from those due to differences in anatomical or physiological assumptions [61]. The report provides standardized values for the constituent parts and functions of an arbitrary average man and is divided into three major sections: anatomical values (e.g., organ weight, volume, specific gravity and volume of blood), elemental content and physiological data. A subsequent report, “Basic anatomical and physiological data for use in radiological protection: reference values” moves beyond an emphasis on a “reference man” and presents a series of reference values for both male and female subjects of six different life stages [62].

### 2.2. Specific Exposure Factor Data Resources Presented in Greater Detail

Several resources are available that are focused on a specific type of exposure factor, for example those related solely to human activity patterns, child non-food mouthing behavior or housing information. The data in these resources are typically presented in greater detail than is found in the coverage of the same topic within general exposure factors compendia.

#### 2.2.1. Human Activity Patterns

Understanding how and where people spend their time is an important part of understanding exposure. This information is needed to determine what environmental media people are likely to come into contact with (air, water, etc.), activity-dependent intake rates of media (e.g., inhalation rates are higher during physical activity), other exposure sources that may be present (e.g., residential products or objects) and housing characteristics that can impact exposure potential (e.g., residential or workplace ventilation patterns and room sizes). Summary information on activity patterns is available in compendia listed in Section 2.1, but often, more detailed information can be useful to improve the exposure assessment, either by focusing on specific microenvironments or enabling probabilistic analyses. Databases and other resources specific to human activity patterns are listed below. The databases included in this section cover human activity patterns in general. They provide information broadly applicable for estimating human exposure, for example activity pattern data, such as time indoors vs. outdoors or in residence vs. school; consumer-product-specific habits and practices are discussed in a separate section below.

- The U.S. EPA Consolidated Human Activity Database (CHAD) (2000, 2010) compiles detailed data on human behavior and activity patterns from separate pre-existing U.S. human activity pattern studies and presents the data in a consistent format [63]. Two versions are available, CHAD-2000 containing detailed information on early studies, and CHAD-Master, which is the most current version and contains information from 22 studies performed during the period 1982–2010. The database includes more than 54,000 individual study days of detailed human behavior, with each day broken down into individual hours and activity types. The database also includes demographic information, such as age, sex, employment and education level, which allows researchers to examine specific groups within the general population and how their unique behavior patterns influence their exposures to chemicals. It is available for download in comma-separated value (CSV) and Statistical Analysis System (SAS) dataset formats.
- The American Time Use Survey was initiated in 2003 and has continued annually, with over 159,000 interviews conducted between 2003 and 2014 [64]. This survey is designed to provide nationally-representative estimates of how and where Americans spend their time. Summary reports, charts, user-support documents and microdata files are available online.
- The Multinational Time Use Research Database includes links to time use activity data for surveys from 22 countries [65]. Datasets are available in a harmonized form and/or aggregate form depending on the survey. Multiple surveys are available for some countries. The surveys range from the years 1965–2015 depending on the country and are available from the Centre for Time Use Research at the University of Oxford. Access to the main data requires registration, and access to some included datasets is further restricted and requires additional authorization.
- “Age Determination Guidelines: Relating Children’s Ages to Toy Characteristics and Play Behavior” from the U.S. Consumer Product Safety Commission (CPSC) addresses the physical, cognitive, emotional and social development of children to identify the types of toys parents and others would purchase for children and how the children interact with those toys [66]. The information in this report can be used to better understand the types of toys that children are likely to play with at a given age.
- “Consideration of Age-Related Changes in Behavior Trends in Older Adults in Assessing Risks of Environmental Exposures” (2013) explores age-related differences in activity patterns and provides activity patterns for older U.S. adults [67]. The authors utilized information in the U.S. EPA EFH, U.S. EPA Consolidated Human Activity Database and peer-reviewed literature.

In addition to the databases listed above, information can also be found from Internet searches of specific activities. For example, for the outdoor activity of camping, an Internet search yielded reports, such as the Outdoor Foundation report on U.S. camping activities, which includes data on the percent of population that camps, days/year camping and favorite camping activities [68]. Information identified in this manner can be helpful for informing an assessment, but can also yield multiple studies of varying design that often may not have gone through a peer review process. Studies should be evaluated for representativeness to the population of interest and data quality in general before the data are used in assessments.

#### 2.2.2. Child Non-Food Mouthing Behavior

Mouthing of non-food items (toys, clothing, fingers, etc.) can be an important exposure consideration, especially for children. The frequency and duration of mouthing activities are both important parameters, as well as the type of activity (hand-to-mouth or object-to-mouth and, if the latter, what type of object). Data from multiple studies are summarized in the U.S. EPA EFH and the RIVM “Children’s Toys Fact Sheet”, and some are also included in ExpoFacts [30,38,69]. These references include a number of specific studies, which can be consulted for detailed information. A source of variability between studies is that definitions of object categories vary across studies (i.e., different studies report mouthing times for categories of items that may or may not have the same individual items) [70]. When applying this information, it is thus important to consider the basis of the reported mouthing time. Observational studies for mouthing activity are available for the U.S., E.U. and Asia regions.

- For the U.S., the EFH indicates key studies for mouthing duration, including Juberg et al. (2001), Greene (2002) and Beamer et al. (2008) [71,72,73]:
  ○ Juberg et al. (2001) was a phased study [71]. In Phases 1 and 2, parental observations were collected for 107 children aged 0–<18 months and 110 children aged 19–36 months each for a one-day period for the type and duration of each item mouthed. In Phase 3, 168 children were each observed for five nonconsecutive days over a two-month period for total mouthing time of objects exclusive of pacifiers. Results are provided for the categories of pacifier, teether, plastic toy and other objects [71].
  ○ Greene (2002) represents one of the most detailed studies of mouthing available [72]. U.S. children of age 3–36 months were each observed for a total of four hours (*N* = 169). Mouthing time includes time in child’s mouth or touching lips. Daily mouthing times were estimated as hourly mouthing time multipled by totals hours awake and not eating. Mouthing observations are available by specific object types; categories include: all objects, pacifiers, non-pacifiers (subcategories of soft plastic items, not food contact; soft plastic toys, teethers and rattles; soft plastic toys; soft plastic rattlers; other soft plastic items), soft plastic food contact items, anatomy, non-soft plastic toys teethers and rattles, other items (including non-soft plastic food contact items—tableware, drinking cups, bottle nipples), furniture, clothing and miscellaneous items. Additional information on mouthing behavior studies sponsored by the Consumer Product Safety Commission can be found on the CPSC website [74,75].
  ○ Beamer et al. (2008) observed microactivity patterns of 23 farmworker children aged 6–27 months [73]. Each child was videotaped for 2–6 h in their home. Information on the frequency of mouthing and duration per mouthing event were reported. Categories included animal, body/head, clothes/towel/washcloth, fabric (wall/furniture), floor (asphalt/sidewalk, carpet/mat, dirt, rock/brick floor, tile/linoleum floor, wood floor), food (food container/other food, sticky food, water/beverage), footwear, hands or mouth, metal (wall/furniture, tool/appliance), non-dietary water (pool, puddle), paper/wrapper, plastic (wall/furniture, tool/appliance), rock/brick (wall/furniture), toys (fabric, hard, porous plastic, wood), vegetation (grass, vegetation) and wood (tool/appliance, wall/furniture).
- For the E.U., the RIVM “Children’s Toys Fact Sheet” [69] includes data from Groot et al., 1998, and Juberg et al. (2001) (discussed above). An additional and somewhat more recent study is Norris and Smith (2002):
  ○ Groot et al. (1998) collected parental observations for 42 children aged 3–36 months living in The Netherlands [76]. Each child was observed for 15-min periods 10-times per day over two days. Results are provided for the categories of dummy (pacifier), fingers, non-toys, toys for mouthing and other toys. Total mouthing duration is provided. Observations on time spent sucking/biting vs. licking were also made.
  ○ Norris and Smith (2002) reported parental observations of 236 U.K. children aged 1 month–5 years [77]. Each child was observed for 20 sessions of 15 min each over a two-week period. Observations were not requested to be done to cover a specific day, but were split as follows: four weekday and one weekend observation for each of the following periods—waking to 11 a.m., 11 a.m.–2 p.m., 2 p.m.–6 p.m., 6 p.m. and going to bed. Results are presented for total mouthing time, and categories of dummy (pacifier), fingers, toys, other objects and not recorded. Observations on whether mouthing time was licking, sucking or biting are also reported. Furthermore, all specific items recorded as mouthed are provided along with the number of times they were reported as mouthed.
- For Asia, one detailed study is available:
  ○ Sugita et al. (2003) provide results of a mouthing observation study of 25 Japanese children aged 6–10 months [78]. Children were observed 15 min/waking hour over two days. Results are presented as total mouthing time with or without pacifiers. Categories in the study included toys, plastics other than toys, finger/body parts, others (cloth, paper, etc.) and pacifiers. Time by category is visualized in Figure 1 of the article [78].

For mouthing frequency, the EFH recommendations are based on the meta-analyses of Xue et al. (2007) for hand-to-mouth and Xue et al. (2010) for object-to-mouth [70,79]. U.S. EPA has published several meta-analyses of children’s mouthing behavior to aid in estimating incidental, non-dietary ingestion exposure [70,79,80]. Hand- and object-to-mouth frequencies are presented as a function of age group (using standardized EPA age groupings) and location (indoors vs. outdoors). The study authors of those studies found that children who tend to mouth objects do not necessarily have a tendency to mouth hands, and while a statistical difference was found among the various studies for object-to-mouth frequency, no difference was observed for hand-to-mouth frequency, suggesting that methodological differences may affect the results. Xue et al. (2010) suggest that interstudy differences in object-to-mouth frequency are likely related to differences in the definitions of object categories [70]. More information on individual studies on mouthing frequency can be found in the U.S. EPA Exposure Factors Handbook [30].

The following databases include mouthing duration and/or frequency data from one or more of the studies described above:

- The HESI Residential Exposure Factors database was developed by Infoscientific.com, Inc. (Manassas, VA, USA) for Health and Environmental Sciences Institute (HESI) [81]. It includes tables of data on mouthing activity. The database provides ready access to mouthing data in one common format and also includes information on child finger and palmar surface area. It was last updated in 2004. The database can be downloaded free of charge.
- The E.U. ExpoFacts [38] database (described in Section 2.1) includes information from the Groot et al. (1998) study of children in The Netherlands and the Consumer and Competition Policy Directorate (DTI) 2002 study of children from the United Kingdom [76,77].

#### 2.2.3. Housing

Housing information is important for estimating exposures in indoor environments. The exposure potential of substances used, emitted or otherwise present indoors will depend on factors such as room volume and ventilation. Information and recommended default values for housing characteristics (e.g., surface areas, volumes) and transport and loss processes (e.g., air exchange rates, filtration and ventilation systems) for U.S. and E.U. homes are discussed in the RIVM General Fact Sheet, E.U. ExpoFacts Sourcebook and U.S. EPA EFH (all previously discussed in Section 2.1) [30,38,44,45]. In each of these resources, key information relevant to housing factors from detailed, previously-published studies or publicly-available surveys has been extracted and tabulated; for example, Tables 19–26 of the U.S. EPA EFH present the distributions of residential air exchange rates by climate region and season from Murray and Burmaster (1995) [82]. These detailed studies can be further mined for additional information. Several references provide information on ventilation rates associated with garages (Batterman et al., 2006; Emmerich et al., 2003; Graham et al., 1999), which can be considered for tasks and products that are more likely to take place in garage locations, rather than general dwelling rooms [83,84,85].

Additional sources of information that can be consulted include the National Institute of Standards and Technology (NIST) CONTAM multi-zone indoor air quality model [86]. This model incorporates data from two residential housing surveys, the U.S. Energy Information Administration Residential Energy Consumption Survey (RECS) and the U.S. Census Bureau American Housing Survey (AHS) [87,88]. Characteristics addressed include age of dwelling, surface areas of flooring, number of floors, type of foundation and presence of garage. The data from the residential surveys, along with important model parameters, can be mined from the NIST CONTAM model input library files.

Limited information on some residential default values relevant to consumer product use scenarios (e.g., surface area covered with product, volume of bathroom) is contained in the U.S. EPA’s “Standard Scenarios for Estimating Exposure to Chemical Substances during Use of Consumer Products” Volume I and Volume II [89,90]. This two-volume set, however, is quite old (1986), and a comparison with the more recent information provided in the general references above to determine representativeness for current conditions should be considered before use.

### 2.3. Exposure Factors Related to Consumer Product Type and Product Use Patterns

While information on use patterns and behaviors associated with consumer products has increased, information sources remain scattered. Some information has been assimilated into reference documents (e.g., U.S. EPA EFH, RIVM Fact Sheets), yet much of the recent information must be searched for individually [30]. Sources include peer review literature, trade association compendia, product specifications, do-it-yourself use instructions and product packaging use guidance. In addition, recent activities in Europe include the development of Specific Consumer Exposure Determinants, which include detailed information on use patterns by product type that can be used as exposure model inputs (International Association for Soaps, Detergents and Maintenance Products (AISE), 2015; Downstream Users of Chemicals Co-ordination group (DUCC), 2014; Concawe, 2014) [91,92,93] Information sources are organized below by consumer product category based on the system employed by the NIH Household Products Database [94], as this is generally how the information is made available and also how it is applied. Table 2 provides key references for each consumer product category listed.

In the absence of other data, Internet searches can return informal feedback on consumer behavior, for example how often some activities are performed. It is noted that this type of information is not representative of the general population and can be biased. Data quality is unknown. It is possible, however, that future mining of this type of information might be used not in place of, but perhaps to extend the more rigorous information provided by scientific studies or surveys. For example, a study may be available reporting survey results for the frequency of a certain activity (e.g., lawn mowing) for a given population. If information in an Internet search for a different region indicates responses consistent with those of the detailed survey, it may help in deciding if the survey information could be used as a reasonable approximation for the second region in the absence of more specific data.

#### 2.3.1. Personal Care Products: Cosmetics

The RIVM “Cosmetics Fact Sheet” includes information on 35 categories of personal care products, including products used for hair care, bathing and showering, skin care, make-up and nail care, deodorant, oral hygiene, foot care, fragrances, men’s cosmetics, including shaving cream and aftershave, sun cosmetics, baby care products and a miscellaneous category that includes depilatories, essential oils and face paint [95]. Information is given on representative compositions and uses of products within a product category. Models and default parameter values for all 35 product categories are provided to aid in assessing the exposure and intake of compounds in cosmetics for men, women and children.

The Cosmetic Ingredient Review was established in 1976 by the Cosmetic, Toiletry and Fragrance Association (CTFA) (now known as the Personal Care Products Council (PCPC)) to review and assess the safety of ingredients used in cosmetics [96]. A formal review process is used to determine with reasonable certainty if an ingredient is safe under its conditions of use. Through regular expert panel meetings, families of substances (e.g., ceramides) are evaluated. The resulting documents, available on the panel meetings website, include information on the types of products in which the substances are used, the typical concentrations within those products and the amount and frequency of product use.

Additional information on exposure models, exposure factors and ingredient concentrations for formulated personal care products for the U.S, Europe and Asia is available in several journal articles [97,98]. Results from a study of the “Habits and Practices” (both the amount of cosmetic product applied and the frequency of use) of female U.S. consumers conducted by the Personal Care Products Council (CTFA/PCPC) in 2000 are available for lipstick, body lotion and face cream (2005), hairspray, spray perfume, liquid foundation, shampoo, body wash and solid antiperspirant (2006) and facial cleanser, hair conditioner and eye shadow (2008) [21,22,23]. Three hundred and sixty women, who defined themselves as regular cosmetics users and were between the ages of 19 and 65 years, were recruited for this study from ten geographically-dispersed locations within the U.S. For each product type of interest, participants were provided with a new container of the brand they normally use and were asked to keep diaries and record detailed daily usage information over a two-week period. Products were also weighed to determine the total amount used over the study period [21,22,23].

Similar information for European consumers is available for a smaller number of product categories (namely body lotion, deodorant/antiperspirant sprays and non-sprays, lipstick, facial moisturizer, shampoo and toothpaste), but from a much larger study population in Hall et al. (2007) [99]. The information on the habits and practices of consumers in the European Union was collected by the European Cosmetics Association COLIPA (now Cosmetics Europe) in a survey of 44,100 households and 18,057 individual habitual users of cosmetics. These data were combined with usage data from an additional 496 individuals. A significant finding from this study was that for body lotion, facial moisturizer, toothpaste and shampoo, there appears to be an inverse correlation between the frequency of product use and the quantity used per application event. Data from this publication and the three Loretz publications are also available in the U.S. EPA EFH, along with additional data from U.S. studies [21,22,23,30].

In the more recent second phase of the study reported by Hall et al. (2007), exposure data in terms of daily quantity (g/day and g/kg/day) of product use for five additional cosmetic product types is presented in Hall et al. (2011) [25,99]. Use information for hair styling, hand cream, liquid foundation, mouthwash and shower gel was obtained from a total of 80,000 households and 14,413 individual consumers in five European countries. These data from COLIPA/Cosmetics Europe have been combined with the data in the RIVM Cosmetics Fact Sheet by the European Commission Scientific Committee on Consumer Safety (SCCS) to estimate daily exposure levels among the European population for the most commonly-used cosmetic product types [100]. Specifically, the SCCS Notes of Guidance for the Testing of Cosmetic Substances and their Safety Evaluation provides default values for skin surface area involved, frequency of application, daily amount applied, retention factors and daily exposure (g/day or mg/kg bw/day) for an assortment of personal care product types.

To address an information gap on personal care product use among European children, Manová et al. (2013) investigated the prevalence and frequency of use in Switzerland of eight commonly-applied “leave-on” product types [29]. The product types selected were face cream, body lotion, aftershave lotion/balm, hand cream, makeup foundation, lip care, lipstick and sunscreen. Information was collected via a self-administered questionnaire with a final sample size of 397 children and adolescents and 799 adults for completed surveys. In an effort to extend beyond merely the frequency and amount of product used, Biesterbos et al. (2013) also investigated the “circumstances of use”, including indoor or outdoor location and the presence of ventilation, of 32 personal care product types among over 500 participants in The Netherlands [28].

Expanding to Asia, Park et al. (2015) collected exposure factor information from 2500 households throughout Korea in 2012 [101]. Their purpose was to develop a database of exposure factors for aggregate consumer exposure assessment. To do so, they conducted face-to-face interviews about the use of five personal care products (face cleanser, toothpaste, shampoo, hair conditioner and body wash), as well as five home care products (dish detergent, laundry detergent, fabric deodorizer, anti-static spray and shoe polish). Various techniques (such as a card with five differently-sized circles for cream, lotion and foam products) were used to obtain information on the amount per application or use event. A comparison of the product use rate and frequency to that in The Netherlands and in the U.S. is provided.

In the U.S., recent information on the use of personal care products has been generated from the EPA-funded Study of the Use of Products and Exposure-Related Behavior (SUPERB) [102]. Usage patterns of 30 types of personal care products were collected from 604 California households through a telephone interview and reported by Wu et al. (2010) [24]. Use frequency data are stratified by age groups and socio-demographic characteristics, and product co-use is quantified using correlation coefficients. In a different investigation by the same group, Bennett et al. (2012) visited 47 California households and tracked the use of 17 categories of personal care products (including antibacterial soap, hair styling products and baby care products), as well as six categories of cleaning products [26]. The investigators used a variety of techniques to minimize participant burden and potential recall bias, including barcode scanning, product weighing and participant dispensing. Comparisons of use frequencies and per-use amounts with data available in the U.S. EPA EFH are presented in both of these publications [30].

Information on the surface areas of body parts onto which personal care products are applied can be obtained from both the U.S. EPA EFH [30] and the RIVM “Cosmetics Fact Sheet” [30,95]. More specific, measured data on the surface areas of underarms of both men and women is available from Cowan-Ellsberry et al. (2008) [103].

#### 2.3.2. Children’s Toys

Young children can face elevated exposures to residential chemicals not only because of their developmental physiology, but also because of child-specific behaviors, such as mouthing of toys. The RIVM “Children’s Toys Fact Sheet” defines 17 different exposure categories related to interaction with toys and provides at least one representative example for each [69]. It includes general information on child mouthing, hand-mouth contact activities and skin contact. It also includes information (including frequency and duration of contact) specific to child products, such as cuddly toys, face paint, finger paint, modeling clay and felt pens. As with all of the RIVM Fact Sheets [44,45], a quality factor (between one and nine) is supplied for all parameter values, indicating the reliability of the estimate of the default value. The general information presented earlier on non-food mouthing is also relevant for the assessment of exposure via toys.

#### 2.3.3. Air Fresheners

Use patterns from a Belgian survey of users of air fresheners are reported in Torfs et al. (2008) for seven types of air fresheners: gel, scented candle, candle, spray, liquid air freshener, electric diffuser and incense [104]. The Torfs document also reports the results of studies of emission factors. For some types of air fresheners, such as manual spray, consumer use patterns are significant determinants of exposure potential. For other types, however, such as electric diffusers (plug-ins), emissions are automated. For these latter types of products, in some cases, manufacturers’ online specifications on product lifetime can help in estimating exposure potential. For example, the daily emission rate of a plug in air freshener can be estimated from manufacturer information on product lifetime (daily emission = total product weight/days in lifetime).

#### 2.3.4. Household Cleaners

Standard scenarios for assessing consumer exposure to chemicals in household cleaners are found in Volume I of U.S. EPA’s Standard Scenarios publication [89]. The document identifies the exposure routes and pathways for each scenario and includes values for all parameters needed to estimate human exposure. Floor wax/polish and antistatic sprays, laundry detergent and softener and dishwashing detergents are also included, in addition to household cleaners. This document also includes estimates of the weight fraction based on the functional category, which can be useful for refining exposure estimates. The age of this document (1986) should be considered if used for contemporary exposure assessments.

A table of common practices of Western Europe populations with respect to the use of laundry detergents and additives, fabric conditioners, dishwashing detergents (hand and machine), surface cleaners and toilet cleaners is readily available. The “Table of Habits and Practices for Consumer Products in Western Europe” was developed by the International Association for Soaps, Detergents and Maintenance Products (AISE) within the European Human and Environmental Risk Assessment on ingredients of household cleaning products (HERA) project in 2002 (and amended by AISE. with additional product categories in 2009) and is available as a download from the AISE website [91]. The table is also incorporated in to a 2005 Guidance Document from HERA titled: Methodology of Risk Assessment in HERA [105]. This document supports the assessment of risks to human health and the environment from ingredients of household cleaning products from both household use and disposal. It does so by providing the necessary details of the procedure for performing the risk assessments.

AISE also recently released Specific Consumer Exposure Determinants fact sheets providing defaults values and their basis for scenarios, including laundry products, fabric conditioners, non-spray surface cleaner, spray liquid surface cleaner, auto dishwashing products, hand dishwashing liquid, non-spray and spray polishes and wax blends and non-aerosol and aerosol air care products in the home (AISE 2015) [91].

To obtain similar information on the habits and practices for the North American population, the Soap and Detergent Association (now the American Cleaning Institute) commissioned a product survey of its member organizations. The survey included nine laundry product types, three dishwashing product types and seven general cleaning product types. Information on product use frequency and amount, as well as skin contact area and dermal retention, are available in a document titled “Consumer Product Ingredient Safety: Exposure and Risk Screening Methods for Consumer Product Ingredients” that is available from the American Cleaning Institute [106]. Much of the habits and practices information from this document has since been published in Sanderson et al. (2006) [107].

Three documents are available from RIVM that provide exposure factors for cleaning products (aimed at the Dutch population). “Hygienic Cleaning Products Used in the Kitchen: Exposure and Risks” provides necessary parameters for assessing exposure to chemicals in dishwashing liquids, cleaning wipes, spray cleaners and bleach containing products (including abrasive, all-purpose cleaner and liquid bleach) [108]. The “Cleaning Products Fact Sheet” covers 36 product categories, including laundry detergents, dishwashing products, abrasives and toilet cleaners, providing default values for all 36 product categories [109]. The “Disinfectant Products Fact Sheet” includes general information on algae and mold removers, indoor disinfectants, swimming pool disinfectants, waterbed conditioners, chemical toilet and rubbish bin disinfectants, veterinary hygiene biocidal products and drinking water disinfectants [110].

In the scientific literature, Hakkinen et al. (1991) focuses on how data on human body weights and surface areas relevant to household cleaning product use are obtained [111]. The review article provides references for a number of industry-sponsored studies of consumer use characteristics. Dimitroulopoulou et al. (2015) present the results of a recent multi-country European survey on the use of 15 household consumer products mainly comprised of cleaning products (but also including some spray-formulated personal care products) [112]. The study provides information not only about the frequency and quantities of product use, but also on the prevalence of use, the location where the products are used and on the ventilation conditions during use. Results are stratified by four geographic locations and by population group (i.e., housekeepers and retired people).

From the SUPERB study described above under “Personal Care Products”, Moran et al. (2012) investigated the consistency over time in use patterns of cleaning products and air fresheners, as well as in the frequency of performing different types of cleaning tasks, among 612 California households from 2006–2009 [27]. The number of users and the number of uses per month for various cleaning products and tasks are presented by household type (older adults versus parents with young children), gender, ethnicity and education level. Previously, Zota et al. (2010) examined cleaning product use frequency in Cape Cod, MA, U.S., among 413 women diagnosed with breast cancer and 403 controls between 1999 and 2000 [113]. Telephone interviews were used to obtain information about five categories of cleaning products, including solid and spray air fresheners, surface cleaners, oven cleaners and mold/mildew products.

#### 2.3.5. Automotive Care

Several literature sources provide exposure scenarios or data on use patterns for automotive care products. Default exposure scenario values for automotive paste wax, upholstery cleaner and fabric protector are provided in Volume I of U.S. EPA’s Standard Scenarios document [89]. Defaults for motor oil, lubricating grease and brake fluid scenarios are provided in Volume II [90]. Data for use patterns for solvent-based automotive products are also provided in the Westat National Household Solvent Survey, but this should be used with caution to represent current conditions given the changes in vehicular design that have taken place over this period [18]. The document includes information on frequency, duration and amount of product per use for the categories of carburetor cleaners, engine degreasers, aerosol spray paint for cars, auto spray primers, spray lubricant for cars, transmission cleaners, battery terminal protectors, brake quieters/cleaners, gasket removers, tire/hubcap cleaners and ignition and wire dryers.

More current scenarios for lubricants and refueling vehicles (auto and recreational) are provided in the Specific Consumer Exposure Determinants (SCED) documents developed by Concaweto support the exposure scenario developed for REACH registrations. Where exposure parameters have been refined as compared to default values, data and references are provided to substantiate the enhancements. These references can be further probed for more information. Results of a recent survey performed to gain greater insights into consumer refueling behaviors for both vehicles and garden equipment are also available [114].

#### 2.3.6. Pesticides: Pest Control Products

The RIVM “Pest Control Products Fact Sheet” provides parameters for assessing exposure and uptake for products that are available to the consumer for private use, namely spray, strips and cassettes, electrical evaporators, insect repellents, baits, dusting powders, textile biocides and gases and foggers [115]. The basis of the default parameter values is to quantify realistic worst-case scenarios, considering consumers who frequently use a certain pest control product under relatively less favorable circumstances. The parameter values are chosen so that their combination represents the 99th percentile of the distribution of exposure and uptake among the Dutch population. A large number of parameters related to the user (e.g., contact time), the product (e.g., mass generation rate) and the conditions of use (e.g., treated surface area) are provided. The fact sheet proposes default models and default parameter values for all product categories covered with the caveat that additional data should be taken into consideration where available.

The U.S. EPA (2012) has recently updated its “Standard Operating Procedures for Residential Pesticide Exposure Assessment” (Residential SOPs), which are designed for estimating exposures from the most common residential pesticide uses [116]. The SOPs provide default methods (algorithms) and parameter values for assessing exposure, both during handling of pesticides and post-application residential activities leading to contact with pesticide residues. SOPs are available for several scenarios, including lawn and garden care, foggers and misters, indoor surface treatments and pet treatments. The current 2012 version of each SOP includes distributional analyses in addition to more simple point estimates, contains exposure factors from the U.S. EPA EFH and is available from the “models and databases” section of the U.S. EPA Office of Pesticide Programs website. Moreover, a series of spreadsheets that are useful for performing the calculations called for in the SOPs are also provided on the website.

#### 2.3.7. Landscape/Yard

A scenario for garden equipment refueling with gasoline is available in the Concawe SCEDs documents, described under the automotive care products section [93]. The U.S. EPA (1999) also has provided generic guidance and exposure factors for residential use of lawn care products in the documentation of its Residential Exposure Assessment (REx) model [117]. This guidance addresses dermal and inhalation exposure during adult application, post application incidental dermal and inhalation exposure for adults and post application incidental dermal, inhalation and oral exposure for children. The more recent U.S. EPA Residential SOPs also include sections on lawn/turf, gardens and trees, and outdoor fogging/misting systems [116].

#### 2.3.8. Home Maintenance

There are a number of sources of information related to habits and practices for home maintenance or “do-it yourself” (DIY) types of products. The U.S. EPA EFH [30] includes summary information from some of the key U.S. surveys:

- The Westat (1987a) Household Solvent Products survey provided information on frequency, amount and some use conditions (e.g., time, location, ventilation) for 32 types of solvent products, including DIY categories, such as degreasers, adhesive removers, paints, wood stains, paint thinners and removers and primers [18].
- Abt, 1992, provided information on frequency, use amount and time for adhesive removers, spray paint and paint removers [118].
- The Westat (1987b) study of interior painters similarly provided information on frequency, time, amount of paint used and protective measures taken for latex paint, oil-based paint and wood stains and varnishes [19].

Other sources include information on default models and/or values for model parameters for DIY products. The RIVM Do-It-Yourself Products Fact Sheet includes information on glues, sealants, fillers and putty, plasters and equalizers, coatings, removers and a miscellaneous category, including insulation foams and joint color [119]. A separate fact sheet is available for paint products that includes information for solvent-rich, high solid and waterborne paints [120]. Volume I of the U.S. EPA’s Standard Scenarios includes defaults for scenarios for residential painting [89]. Volume II provides defaults for exposure scenarios for multiple types of adhesives, foam insulation, wood putty, bathtub caulk and pre-mixed spackling [90]. Related habits and practices information is available in the Westat National Household Solvent Survey [18]. The RIVM “Paint Products Fact Sheet” includes information for solvent reach, high solid and waterborne paints and considers both brush/roller painting, as well as spray paint [120]. As above, there are a number of Internet DIY sites that provide information on use patterns or specifications for do-it-yourself type product use.

#### 2.3.9. Arts and Crafts

Limited information is currently available on exposure factors related to arts and crafts. Volume II of the U.S. EPA’s Standard Scenarios (Versar, 1986b) provides defaults for hobby scenarios, including multiple types of adhesives, photography chemicals, paint remover, wood finishing and refinishing, lacquer thinner and fabric dyes [90]. RIVM fact sheets for toys and do-it-yourself products provide information for scenarios including modeling clay and finger paint, glues from tubes, bottled glue, super glue and hot melt adhesives [69,119]. Other readily available documents tend to focus on safety practices to decrease the risk of illness or injury rather than on exposure factor-related information. Some manufacturer websites provide product specifications that can be used to help in estimating use patterns or may provide additional information upon request. For example, in conducting an assessment of a substance present in one hobby model paint, the manufacturer was contacted and provided information on typical container sizes and the amount of product needed to complete one model. Manufacturer-supplied information can be useful, but remains time consuming to search on an individual basis.

## 3. Using Exposure Factors in Consumer Exposure Assessments

In order to effectively develop a useful exposure estimate, information on individual exposure factors must be combined in a manner that is relevant for the conditions of exposure. The complete set of parameters that help to describe exposure represents the exposure scenario. An exposure scenario includes factors related to product use (amount, frequency, duration), environmental characteristics (room volume, air exchange rate for a residential environment) and physiological factors of the individual being assessed (body weight, inhalation rate, etc.).

To help design a representative consumer exposure scenario, the first step needed is problem formulation (consistent with Pastoor et al., 2014; Embry et al., 2014) [121,122]. That is, an understanding of the population of interest and factors that are consistent with how that population uses and may be exposed to substances in consumer products. Templates for what parameters are relevant for a consumer exposure scenario are available from ECHA guidance documents, from OECD reports, SCEDs guidance and may also be developed by following existing compendia or models [92,123,124]. Guidance on methods for assessing consumer exposure and/or developing consumer exposure scenarios to chemical substances is available from several sources [123,125,126].

In developing a scenario, it is important to consider that the combination of all parameters results in an estimate that meets the objectives of the assessment (i.e., may be designed to give an estimate that represents a worst case, average, range, realistic value for a given subpopulation, etc., depending on the question being addressed). This requires that interrelations of factors be considered. For example, for a do-it-yourself product, such as wall paint, use amount will depend on the room size. Locations may also be product dependent (for example, vehicle refueling operations would take place outdoors, whereas lawn equipment refueling may occur outdoors or in a garage). Product handling may also vary depending on the form (for example, different product use amounts may be associated with liquid as compared to granular laundry detergent). Physiological factors, such as skin surface area and inhalation rate, may be related to body weight, age and/or gender of the population of interest. Because it is the combination of individual parameters that results in the overall exposure scenario prediction, it is important to consider that a given target objective for the scenario (i.e., average, worst case, etc.) does not apply to each of the individual parameters, but to the combination of parameters as a whole. For example, a small room size is often selected to develop a more conservative exposure estimate, as it results in lower air dilution. If, however, the product use amount depends on the room dimensions, such as for a wall paint, the mass of product used should be adjusted to correspond to the room wall surface area. The upper percentile mass values are likely to be associated with use in larger sized rooms. The dependence between individual parameters therefore needs to be considered and maintained during scenario development. Thus, the values of individual parameters may differ in their relative levels of conservatism as compared to the objective for the scenario. The resulting output of the scenario as a whole should match the assumed target objective. If possible, benchmarking of scenario performance by comparing to a similar situation with measured data can be helpful.

Examples of consumer product scenarios are found in several references. U.S. EPA’s “Standard Example Exposure Scenarios” includes an example for dermal contact with consumer products [127], and 44 scenarios for eight product categories are included in the U.S. EPA’s Standard Scenarios document [113]. ECHA (2011) produced a cleaning product example scenario [123]. In addition, scenarios for multiple product types are found in the Specific Consumer Exposure Determinants and in modeling tools like [91,92,93]: the U.S. EPA’s Exposure and Fate Assessment Screening Tool Version 2014 [128], ConsExpo [129], the Targeted Risk Assessment tool (TRA) [130,131,132,133], Chemical Safety Assessment and Reporting tool (CHESAR) [134], REACH Consumer Exposure Assessment Tool (REACT) [135], European Solvents Industry Group Generic Exposure Scenario Risk and Exposure Tool (EGRET) [136,137] and the Wall Paint Exposure Model [138].

Two recent examples of methodologies for using exposure scenarios to model aggregate exposure to ingredients of personal care products can be found in Comiskey et al. (2015) and Tozer et al. (2015) [139,140]. These highlight the usefulness of additional data on product co-use patterns and also compositional information.

## 4. Conclusions

Everyday exposures to chemicals in consumer products and from other sources have been a global discussion topic of public, research, regulatory and legislative interest. The cause of the high level of interest is reflected in a recent report from the U.S. National Academy of Sciences Committee on Human and Environmental Exposure Science in the 21st Century (NRC, 2012), which begins with:

“We are exposed every day to agents that have the potential to affect our health—through the personal products we use, the water we drink, the food we eat, and the soil and surfaces we touch, and the air we breathe.”

[141]

Developing and maintaining data resources are ongoing challenges given potential changes in product formulations, imports and consumer use patterns. Fortunately, efforts to provide information resources have tried to keep pace with the needs, with early noteworthy efforts being the U.S. EPA’s work to compile the first edition of the “Exposure Factors Handbook” in 1989 and then enhanced by the addition in 1997 of chapters on consumer products and housing characteristics and the 2002 release of the “Child-Specific Exposure Factors Handbook”. More recently, both documents have been combined in the 2011 edition of the EFH; these and other updates by the EPA’s Exposure Factors Program can be found at its home page. Links to exposure factor documents from USEPA and other regions are compiled on the ExpoFacts site, to assist the exposure assessor in understanding regional variability.

Exposure factors are far from static. Internationally, new publications and databases providing factors for specific populations with potentially unique characteristics are being released each year. For example, the “Highlights of a Chinese Exposure Factors Handbook” was published in English in 2015. Moreover, factors change over time. The “Canadian Exposure Factors Handbook” documents significant changes in time-activity data over the past two decades as teens, adults and seniors increasingly spend time engaged in indoor sedentary activities. Perhaps not unrelated, the past two decades have also witnessed substantial changes in anthropometric measures among the U.S. population as body weights and related factors, such as food intake, have increased. Housing-related factors, such as housing type and air exchange rate due to infiltration, may also shift, associated with population migration patterns in combination with less predictable factors [142,143].

The vast sources of exposure factor-related information described in this article can serve to complement various efforts aimed at increasing the amount of chemical-specific information available for consumer products. One noteworthy EPA effort in that area is the ExpoCast™ research initiative, which has led to new exposure models and databases, and publications, such as Goldsmith et al. (2014) and Wambaugh et al. (2014) [16,144]. ExpoCast and its related publications are examples of efforts aligned with the National Research Council‘s report on “Exposure Science in the 21st Century” (NRC 2012) support of the development of efficient mechanisms to disseminate data and the application of computational methods to derive new models that better predict exposure.

Improvements in technology will continue to move the science forward, for example the ongoing development of sensors that can be used for personal exposure assessment [145]. The potential for creativity in sensor development is reflected by EPA’s “Village Green” project. It involves the development of benches that will allow people sitting on that bench to use their smartphones to obtain real-time data on the air quality around them. It is envisioned that as these technologies continue to develop, the expanding capability to collect and integrate various types of data can be applied to the residential setting to improve the understanding of current human exposures to consumer product-related chemicals will be greatly enhanced.

A number of the resources identified in this effort are now somewhat dated surveys. Updating this information with current studies would be useful, especially where temporal changes are expected. For example, changes in activity patterns have been noted in the “Canadian Exposure Factors Handbook”, with an increase in indoor sedentary activities over time. It is anticipated that emerging technologies will facilitate the collection of more current data in these areas.

The future looks promising for databases and further development of science and technology relevant to the assessment of consumer exposures. However, monitoring the efforts of governmental organizations like EPA and National Library of Medicine (NLM) in the U.S. and RIVM and ECHA in the E.U., as well as industry trade associations, such as the American Cleaning Institute and Concawe, and other nongovernmental organizations, like HESI, will remain both a challenge and an opportunity. The ability to quickly mine PubMed or other online resource also increases the speed with which new data can be communicated and implemented. Expanding beyond the typical exposure factor resources to examine the utility of large data sources relevant to consumer behavior, such as market surveys and social media, may provide further opportunities to improve exposure factor data. The increased public availability of datasets established for other purposes also provides a new repository to mine for exposure science utility. This type of expansion will require taking on challenges, such as how to ensure data quality and representativeness for a general population. We have found that the development of this document highlights the utility of a systematic compilation, evaluation and review of references for consumer product exposure assessment. Indeed, ideally, a common Internet repository of relevant exposure factor information and/or a consumer product-specific exposure factors handbook that is maintained with current information would be a valuable resource to future consumer exposure evaluations.

## Figures and Tables

**Table 1:** Global general exposure factor compendia and databases by reference population (geography, age, maternity status).

Population	Housing Factors	Time-Activity Patterns	Physiological Factors	Dietary Intake	Non-dietary Ingestion	Consumer Products
North America						
U.S. [30]	✓	✓	✓	✓	✓	✓
U.S. [2]	✓		✓			✓
U.S. [33]		✓	✓	✓		✓
U.S. elderly [34]	✓	✓	✓			
U.S. pregnant [35]		✓	✓	✓		✓
Canada [36]		✓	✓		✓	
Europe						
E.U. [38]	✓	✓	✓	✓	✓	✓
E.U. [41,42]	✓		✓			
Nordic Region [43]		✓	✓	✓	✓	✓
Netherlands [44,45]	✓	✓	✓			
Germany [47]	✓	✓	✓	✓	✓	
Asia						
Korea [48,50]	✓	✓	✓	✓	✓	
Japan [56]		✓	✓	✓	✓	
China [57]	✓	✓	✓	✓		✓
Australia [58]	✓	✓	✓	✓	✓	
INTERNATIONAL						
Multinational [49]		✓	✓	✓		
Multinational [60]			✓			
Nonspecific [61,62]			✓			

**Table 2:** References organized by information type and consumer product category.

	Personal Care	Inside the Home *	Automotive Care	Pesticides/Landscape/Yard	Home Maintenance	Arts and Crafts
Habits and Practices **	[17,21,22,23,24,25,26,28,29,30,95,96,99,101]	[17,26,27,30,104,105,106,108,109,110,113]	[18,30,89,90,93,114]	[30,93,115,116,117]	[18,19,118,119,120]	[69,90,119]
User demographics	[21,22,23,24,29,30]	[10,27,30]	[18,30]	[30]	[18]	
Ingredient Name	[95,96,97,98]	[89,90,97,98,108,109,110]	[94]	[94,115]	[89,90,94,119,120]	[90]
Ingredient Weight Fraction	[95,96,97,98]	[89,90,97,98,108,109,110]	[94]	[94,115,116]	[89,90,94,119,120]	[90]
Ingredient Function	[95,96]	[89,90,108,109,110]		[115]	[89,90,119,120]	[90]

* Includes toys, air fresheners and household cleaners. ** Includes patterns (frequency, amount and duration) and prevalence of use.

## Supplementary Materials

The following are available online at www.mdpi.com/1660-4601/13/7/744/s1, Table S1: Compilation of Cited Hyperlinks. (Organizations can make changes to hyperlinks and an online search for an updated link is suggested if a hyperlink appears to be broken).
