# Supplementary material for: Exploring Global Exposure Factors Resources for Use in Consumer Exposure Assessments
Source: Int J Environ Res Public Health. 2016 Jul 22;13(7):744. doi: 10.3390/ijerph13070744 (PMC4962285; doi:10.3390/ijerph13070744)
Supplement: Supplementary file 1 [file ijerph-13-00744-s001.pdf]

# Supplementary Materials: Exploring Global Exposure Factors Resources for Use in Consumer Exposure Assessments

**Rosemary T. Zaleski, Peter P. Egeghy and Pertti J. Hakkinen**

**Table S1.** Compilation of Cited Hyperlinks. (Organizations can make changes to hyperlinks and an online search for an updated link is suggested if a hyperlink appears to be broken).

| RESOURCE | URL |
|---|---|
| ACI (American Cleaning Institute) | http://www.cleaninginstitute.org/science.aspx |
| ACI Consumer Product Ingredient Safety: Exposure and Risk Screening Methods for Consumer Product Ingredients | http://www.aciscience.org/docs/Consumer_Product_Ingredient_Safety_v2.0.pdf |
| AISE (International Association for Soaps, Detergents and Maintenance Products) | http://www.aise.eu/ |
| AISE/FEA Specific Consumer Exposure Determinants (SCEDS) | https://www.aise.eu/documents/document/20150602150536-aise_sceds_factsheets_may2015_v1.pdf |
| AISE REACT (Reach Exposure Assessment Consumer Tool) | http://www.aise.eu/our-activities/product-safety-and-innovation/reach/consumer-safety-exposure-assessment.aspx |
| American Housing Survey | http://www.census.gov/programs-surveys/ahs.html |
| American Time Use Survey | http://www.bls.gov/tus/ |
| Australian Environmental Health (enHealth) Standing Committee | http://www.health.gov.au/internet/main/publishing.nsf/Content/ohp-environ-enhealth-committee.htm |
| Australian (enHealth) Exposure Factor Guide | http://www.health.gov.au/internet/main/publishing.nsf/Content/health-pubhlth-publicat-environ.htm |
| Australian (enHealth) Environmental Health Risk Assessment Guidelines | http://www.health.gov.au/internet/main/publishing.nsf/Content/A12B57E41EC9F326CA257BF0001F9E7D/$File/DoHA-EHRA-120910.pdf |
| Canadian Exposure Factors Handbook | http://www.usask.ca/toxicology/docs/cef |
| Chinese Exposure Factors Handbook – Adults (Highlights) | https://books.google.com/books?id=wrZ7BgAAQBAJ&source=gbs_navlinks_s |
| CONCAWE Consumer Refueling Behaviors for both Vehicles and Garden Equipment | https://www.concawe.eu/uploads/Modules/Publications/rpt_12-11-2013-00130-01-e.pdf |
| CONCAWE Specific Consumer Exposure Determinants (SCEDS) Documents | https://www.concawe.eu/reach/specific-consumer-exposure-determinants-sceds-documents |
| Cosmetic Ingredient Review (of the Personal Care Products Council) | http://www.cir-safety.org/ |
| Cosmetics Europe (Cosmetics and Personal Care Industry Trade Group) | https://www.cosmeticseurope.eu/ |
| CPSC (Consumer Product Safety Commission) Homepage | http://www.cpsc.gov/ |
| CPSC Age Determination Guidelines: Relating Children's Ages to Toy Characteristics and Play Behavior | http://www.cpsc.gov/PageFiles/112234/adg.pdf |
| DUCC (Downstream Users of Chemicals Co-ordination group) SCEDs guidance | http://www.ducc.eu/documents/20140424-Guidance documents on SCEDs-Final-V1.pdf |
| ECETOC (European Center for Ecotoxicology and Toxicology of Chemicals) Homepage | http://www.ecetoc.org/ |
| ECETOC Exposure Factors Sourcebook for European Populations (with focus on UK data) | http://www.ecetoc.org/technical-reports |
| ECETOC Targeted Risk Assessment Tool (TRA) | http://www.ecetoc.org/tra |
| ECHA (European Chemicals Agency) Homepage | http://echa.europa.eu/ |
| ECHA CheSAR (Chemical Safety Assessment and Reporting tool) | https://chesar.echa.europa.eu/ |
| ECHA Exposure Scenario for Chemical Safety Report and Communication; Example: Consumer Cleaning Products | http://echa.europa.eu/documents/10162/13564/es_for_consumer_20110829_en.pdf |

**Table S1.** *Cont.*

| RESOURCE | URL |
| --- | --- |
| ECHA Guidance Chapter R. 15: Consumer Exposure Estimation | http://echa.europa.eu/documents/10162/13632/information_requirements_r15_en.pdf |
| ECHA Guidance Chapter R. 17: Estimation of Exposure from Articles | http://echa.europa.eu/documents/10162/13632/information_requirements_r17_en.pdf |
| ECHA Guidance on Information Requirements and Chemical Safety Assessment | http://guidance.echa.europa.eu/ |
| ESIG (European Solvent Industry Group) | http://www.esig.org/en/regulatory-information/reach/ges-library/consumer-gess |
| E.U. ExpoFacts Database | http://expofacts.jrc.ec.europa.eu/ |
| HERA Human and Environmental Risk Assessment on ingredients of household cleaning products | http://www.heraproject.com/ |
| HERA Methodology of Risk Assessment | http://www.heraproject.com/files/HERA TGD February 2005.pdf |
| HESI Residential Exposure Factors database | http://hesiglobal.org/hesi-residential-exposure-factors-database/ |
| ICRP Report of the Task Group on Reference Man | http://www.icrp.org/publication.asp?id=ICRP%20Publication%2023 |
| ISES (International Society of Exposure Science) Homepage | http://www.isesweb.org/ |
| Japanese Exposure Factors Handbook | https://unit.aist.go.jp/riss/crm/exposurefactors/english_summary.html |
| Korean Exposure Factors Handbook | http://www.ncbi.nlm.nih.gov/pmc/articles/PMC3930810/ |
| Multinational Time Use Research Database | http://www.timeuse.org/mtus/database |
| NIH (National Institutes of Health) Household Products Database | http://householdproducts.nlm.nih.gov/ |
| NIST (National Institute of Standards and Technology) Homepage | http://www.nist.gov/ |
| NIST CONTAM model input library files | http://www.bfrl.nist.gov/IAQanalysis/CONTAM/libraries.htm |
| NIST CONTAM multizone indoor air quality model | http://www.nist.gov/el/building_environment/contam_software.cfm |
| Nordic Council of Ministers Existing Default Values and Recommendations for Exposure Assessment | http://orbit.dtu.dk/services/downloadRegister/52688829/Exposure_rapport.pdf |
| OECD Assessment of Chemicals Reports | http://www.oecd-ilibrary.org/environment/oecd-series-on-testing-and-assessment_20777876 |
| OECD Guidance Document on Reporting Summary Information on Environmental, Occupational and Consumer Exposure | http://www.oecd-ilibrary.org/docserver/download/9750421e.pdf |
| Personal Care Products Council | http://www.personalcarecouncil.org/ |
| RIVM (Dutch National Institute for Public Health and the Envvironment) Homepage | http://www.rivm.nl/ |
| RIVM ConsExpo Software | http://www.rivm.nl/en/Documents_and_publications/Scientific/Models/Download_page_for_ConsExpo_software |
| RIVM Fact Sheets Homepage | http://www.rivm.nl/en/Topics/C/ConsExpo/Fact_sheets |
| RIVM ConsExpo General Fact Sheet | http://www.rivm.nl/dsresource?objectid=rivmp:266571&type=org&disposition=inline&ns_nc=1 |
| RIVM Children's Toys Fact Sheet | http://www.rivm.nl/en/Search/Library?query=toy+fact+sheet |
| RIVM Cleaning Products Fact Sheet | http://www.rivm.nl/en/Documents_and_publications/Scientific/Reports/2006/augustus/Cleaning_Products_Fact_Sheet_To_assess_the_risks_for_the_consumer |
| RIVM Cosmetics Fact Sheet | http://www.rivm.nl/en/Documents_and_publications/Scientific/Reports/2006/augustus/Cosmetics_Fact_Sheet_To_assess_the_risks_for_the_consumer_Updated_version_for_ConsExpo_4 |
| RIVM Disinfectant Products Fact Sheet | http://www.rivm.nl/en/Documents_and_publications/Scientific/Reports/2006/augustus/Disinfectant_Products_Fact_Sheet_To_assess_the_risks_for_the_consumer |
| RIVM Do-It-Yourself Products Fact Sheet | http://www.rivm.nl/Documenten_en_publicaties/Wetenschappelijk/Rapporten/2007/juli/Do_It_Yourself_Products_Fact_Sheet_To_assess_the_risks_for_the_consumer |

**Table S1.** *Cont.*

| RESOURCE | URL |
|---|---|
| RIVM Paint Products Fact Sheet | http://www.rivm.nl/en/Documents_and_publications/Scientific/Reports/2007/augustus/Paint_Products_Fact_Sheet_To_assess_the_risks_for_the_consumer_Updated_version_for_ConsExpo_4 |
| RIVM Pest Control Products Fact Sheet | http://www.rivm.nl/en/Documents_and_publications/Scientific/Reports/2006/augustus/Pest_Control_Products_Fact_Sheet_To_assess_the_risks_for_the_consumer_Updated_version_for_ConsExpo_4 |
| RIVM Hygienic Cleaning Products Used in the Kitchen: Exposure and Risks | http://www.rivm.nl/en/Documents_and_publications/Scientific/Reports/1999/oktober/Hygienic_Cleaning_Products_used_in_the_kitchen_Exposure_and_risks |
| SCCS (Scientific Committee on Consumer Safety) Notes of Guidance for the Testing of Cosmetic Substances and their Safety Evaluation 8th Revision. | http://ec.europa.eu/health/scientific_committees/consumer_safety/docs/sccs_s_006.pdf |
| Society for Risk Analysis (SRA) Homepage | http://www.sra.org/ |
| SRA/ISEA Residential Exposure Assessment Sourcebook | http://books.google.com/books?id=dz6EvMSx0QIC&printsec=frontcover |
| SUPERB (Study of Use of Products and Exposure-Related Behavior) | http://superb.ucdavis.edu/content-publications.html |
| U.S. Census Bureau Homepage | http://www.census.gov/en.html |
| U.S. Consumer Product Safety Commission Homepage | http://www.cpsc.gov/ |
| U.S. Energy Information Administration (U.S. EIA) Homepage | http://www.eia.gov/ |
| U.S. EIA Residential Energy Consumption Survey | http://www.eia.gov/consumption/residential/ |
| USEPA and ILSI Databases for Physiological Parameters for PBPK Modeling | http://cfpub.epa.gov/ncea/cfm/recordisplay.cfm?deid=202847 |
| USEPA Office of Chemical Safety and Pollution Prevention (OCSPP) Homepage | https://www.epa.gov/aboutepa/about-office-chemical-safety-and-pollution-prevention-ocspp |
| USEPA Exposure Factors Program home page | http://cfpub.epa.gov/ncea/cfm/recordisplay.cfm?deid=20563 |
| USEPA Exposure Factors Handbook (2011 Edition) | https://www.epa.gov/expobox/about-exposure-factors-handbook |
| USEPA Child Specific Exposure Factors Handbook | http://cfpub.epa.gov/ncea/cfm/recordisplay.cfm?deid=199243 |
| USEPA Example Exposure Scenarios | http://ofmpub.epa.gov/eims/eimscomm.getfile?p_download_id=435481 |
| USEPA Child-Specific Exposure Scenarios Examples | http://cfpub.epa.gov/ncea/risk/recordisplay.cfm?deid=262211 |
| USEPA Data Sources for Modeling Environmental Exposures in Older Adults | https://cfpub.epa.gov/si/si_public_file_download.cfm?p_download_id=508404 |
| USEPA Consolidated Human Activity Database | https://www.epa.gov/healthresearch/consolidated-human-activity-database-chad-use-human-exposure-and-health-studies-and |
| USEPA Exposure and Fate Assessment Screening Tool | https://www.epa.gov/tsca-screening-tools/e-fast-exposure-and-fate-assessment-screening-tool-version-2014 |
| USEPA Residential Exposure Assessment (REx) Model | https://archive.epa.gov/scipoly/sap/meetings/web/html/092600_mtg.html |
| USEPA Office of Pesticide Programs website | http://www.epa.gov/pesticides/ |
| USEPA Standard Operating Procedures for Residential Pesticide Exposure Assessment (Residential SOPs) | https://www.epa.gov/pesticide-science-and-assessing-pesticide-risks/standard-operating-procedures-residential-pesticide |
| USEPA ExpoCast Publications | http://cfpub.epa.gov/si/si_lab_search_results.cfm?fed_org_id=1267&SIType=PR&TIMSType=Journal&showCriteria=0&address=ncct%2Fpublications.html&view=citation&sortBy=pubDateYear&keyword=ExpoCast |
| USEPA Village Green project | https://www.epa.gov/air-research/village-green-project |
| Westat National Household Solvent Survey | http://nepis.epa.gov/Exe/ZyPURL.cgi?Dockey=P100754Q.TXT |

© 2016 by the authors; licensee MDPI, Basel, Switzerland. This article is an open access article distributed under the terms and conditions of the Creative Commons by Attribution (CC-BY) license (http://creativecommons.org/licenses/by/4.0/).
